# Is the Success of Plant Invasions the Result of Rapid Adaptive Evolution in Seed Traits? Evidence from a Latitudinal Rainfall Gradient

**DOI:** 10.3389/fpls.2018.00208

**Published:** 2018-02-27

**Authors:** Marco A. Molina-Montenegro, Ian S. Acuña-Rodríguez, Tomás S. M. Flores, Rasme Hereme, Alejandra Lafon, Cristian Atala, Cristian Torres-Díaz

**Affiliations:** ^1^Centro de Estudios Avanzados en Ecología Molecular y Funcional, Instituto de Ciencias Biológicas, Universidad de Talca, Talca, Chile; ^2^Centro de Estudios Avanzados en Zonas Áridas, Facultad de Ciencias del Mar, Universidad Católica del Norte, Coquimbo, Chile; ^3^Research Program “Adaptation of the Agriculture to Climate Change” PIEI A2C2, Universidad de Talca, Talca, Chile; ^4^Centro de Investigación en Ecosistemas de la Patagonia, Coyhaique, Chile; ^5^Laboratorio de Anatomía y Ecología Funcional de Plantas, Instituto de Biología, Facultad de Ciencias, Pontificia Universidad Católica de Valparaíso, Valparaíso, Chile; ^6^Grupo de Biodiversidad y Cambio Global, Departamento de Ciencias Básicas, Universidad del Bío-Bío, Chillan, Chile

**Keywords:** dandelion, clines, heritability, invasive, latitudinal gradient, reciprocal transplant, seed traits

## Abstract

It has been widely suggested that invasion success along broad environmental gradients may be partially due to phenotypic plasticity, but rapid evolution could also be a relevant factor for invasions. Seed and fruit traits can be relevant for plant invasiveness since they are related to dispersal, germination, and fitness. Some seed traits vary along environmental gradients and can be heritable, with the potential to evolve by means of natural selection. Utilizing cross-latitude and reciprocal-transplant experiments, we evaluated the adaptive value of seed thickness as assessed by survival and biomass accumulation in *Taraxacum officinale* plants. In addition, thickness of a seed and Endosperm to Seed Coat Proportion (ESCP) in a second generation (*F*_2_) was measured to evaluate the heritability of this seed trait. On the other hand, we characterized the genetic variability of the sampled individuals with amplified fragment length polymorphism (AFLP) markers, analyzing its spatial distribution and population structure. Overall, thickness of seed coat (plus wall achene) decreases with latitude, indicating that individuals of *T. officinale* from northern populations have a thicker seed coat than those from southern populations. Germination increased with greater addition of water and seeds from southern localities germinated significantly more than those from the north. Additionally, reciprocal transplants showed significant differences in survival percentage and biomass accumulation among individuals from different localities and moreover, the high correlation between maternal plants and their offspring can be suggesting a high grade of heritability of this trait. Although genetic differentiation was found when was considered all populations, there was no significant differentiation when only was compared the northernmost populations which inhabit in the driest climate conditions. Our results suggest that climatic conditions could affect both, the ESCP and the genetic variability in the invasive *T. officinale*, suggesting that this seed trait could be indicative of adaptive selection. Thus, colonization along broad geographical gradients in many cases may be the result –in part- for the presence of functional traits as shown in invasive plant species with rapid adaptive capacity.

## Introduction

Most of invasive species spread in their non-native environments occupying large geographical areas, resulting in plant populations that experience very different climatic conditions (Rejmánek et al., 2005; Molina-Montenegro et al., 2013). Hence, it is logical to assume that the vegetative and reproductive traits of invasive species must experience post-introduction evolution in order to maximize their performance and establishment in those new environments (Chun et al., 2007; Molina-Montenegro et al., 2010). It has been widely suggested that both colonization and invasion success along broad environmental gradients may be partially due to phenotypic plasticity (Sexton et al., 2002; Geng et al., 2007; Molina-Montenegro et al., 2013). In addition, rapid evolution in several traits related with environmental tolerance or resources up-take could also be a relevant factor for invasions (Colautti and Lau, 2015). Nonetheless, a third possible explanation is related with a high pre-existing genetic diversity in the native range and the later filtering of genotypes that could be locally pre-adapted in the new range (see Colautti and Lau, 2015).

The early studies of Baker (1974) and Brown and Marshall (1981) highlighted the idea that rapid evolutionary changes could be an important driving force for biological invasions. More recently, a growing number of studies have provided substantial evidence showing that exotic species recently introduced to new environments can evolve rapidly (Lee, 2002; Maron et al., 2004; Lavergne and Molofsky, 2007; Dlugosh and Parker, 2008; Franks and Weis, 2008; Schierenbeck and Ellstrand, 2009; Molina-Montenegro et al., 2011; Colautti and Lau, 2015). Thus, rapid genetically based adaptation to novel environments might be more important in the ecology of invasions than previously thought. For example, Chun et al. (2007) showed that the invasive *Lythrum salicaria* (purple loosestrife) exhibited distinct flowering time and flower production characteristics in its introduced range in comparison to its native range, suggesting rapid evolution in several reproductive traits. In addition, Alexander (2013), explaining the invasion success of *Lactuca serriola*, noted the capacity of non-native populations to rapidly produce phenotypic and genetic adaptive variation along the invaded climate gradient. Furthermore, they proposed that this response could be related to its wide native geographic distribution and the consequent environmental variation.

It has been shown that selective pressures may drive contemporary evolution within exotic plant populations in different ways. In particular, abiotic gradients across the introduced range could impose divergent selection and promote genetically based differentiation among introduced populations (see Maron et al., 2004). A classic manifestation of this would be the evolution of geographic clines, as is often found among plant populations occurring across latitudinal (Santamaría et al., 2003; Prendeville et al., 2013) or altitudinal (Molina-Montenegro and Cavieres, 2010; Kooyers et al., 2015) gradients. However, among cosmopolitan plants little is known about whether these clinally distributed traits have adaptive consequences, this is, if they produce differential expressions of fitness depending on the geographic position along a particular abiotic gradient, and if these traits are heritable.

Seed and fruit traits are critical for dispersal syndromes and mechanisms to cope with environmental stress. They are also sensitive to changes in biotic and abiotic conditions (Baskin and Baskin, 2001; Moles et al., 2005). For example, seed coat thickness can be affected by rainfall, and is inversely related to imbibition capacity and germination rate (Baskin and Baskin, 2001). Additionally, germination rate and carbon mobilization in seed are influenced by seed coat thickness, with a lower germination percentage in seeds with thicker coats (Noodén et al., 1985; Gorim and Asch, 2012). However, in some plant species that have to cope with unpredictable climatic events and low amounts of rain, seeds develop a thicker seed coat in order to have the greatest resistance to extreme drought conditions (Gutterman, 1994). Hence, seed germination shows evidence of adaptive evolution as seed coat thickness is well correlated with germination under different amount of rainfall (Gutterman, 1994). Thus, seed coat thickness plays an important role in reducing seedling mortality risk immediately after germination. Although there is also evidence for a negative relation between seed coat proportion and seed mass (Clements et al., 2002), germination usually depends on the duration of moist periods and rainfall where seeds with thicker seed coats will need higher precipitation to germinate. Thus, as seed traits are variable and may be heritable (Mera et al., 2004), they have the potential to evolve by means of natural selection in a given environment, where a particular seed trait confers higher plant fitness.

In the *Taraxacum* agg. (aggregate dandelion complex), the dispersal unit is the achene, a fruit which is formed by a single seed with a differentiated seed coat and endosperm, enclosed in a dry (not fleshy) and hard pericarp. In *T. officinale*, seed coat is fused with achene wall, and it is the achene that is dispersed, acting ecologically as a seed (Tweney and Mogie, 1999). Thus, achene can be considered functionally as the seed for ecological aspects. Seed shape is somewhat similar within the dandelion complex, but populations and ecotypes differ drastically in seed size and coat proportion (Tweney and Mogie, 1999; Honek et al., 2011) with many ecological and evolutionary implications. *T. officinale* is a perennial herb native from Europe and currently considered to be a very aggressive invasive species almost elsewhere outside its native range (Holm et al., 1997), with a mainly apomictic reproduction but sexual reproduction has been also recorded (van der Hulst et al., 2000). *T. officinale* in Chile was initially recorded in the city of Santiago in 1870 (Matthei, 1995) and posteriorly in the southern city of Valdivia (39.8°S) in 1875. Later this species was recorded in northern cities (La Serena and Caldera) in 1947 and 1949, respectively. Finally, the colonization in southernmost city of this study (Coyhaique) has recently in 1988 (for more details see Molina-Montenegro MA; repository of Ph.D. thesis, Universidad de Concepción)^1^. Originary from European alpine environments, *T. officinale* is also widely distributed among different elevations in its native range, particularly associated with disturbed sites (Quiroz et al., 2009; Molina-Montenegro et al., 2011). Contrastingly, in Chile this exotic species has been found growing abundantly in zones with a wide variation in disturbance levels (Cavieres et al., 2005), being majority of populations triploid with 24 chromosomes (Molina-Montenegro et al., 2013). Moreover, *T. officinale* has expanded its southern distributional limit (Molina-Montenegro and Naya, 2012) and has colonized higher elevations in the Andes of Central Chile (Cavieres et al., 2008; Molina-Montenegro and Cavieres, 2010). Considering its latitudinal and altitudinal spread, it can be supposed that its invasive capacity is not limited by local conditions.

In the present study we addressed the following questions: (i) does the mean thickness of endosperm and coat (seed coat plus achene wall), and the average proportion between the endosperm and the seed coat (as considered above) vary between populations of *T. officinale* located along a latitudinal gradient with a marked variation in the rainfall?, (ii) does this variation have any adaptive relevance as assessed by greater germination, survival and biomass?, (iii) are these functional seed traits heritable?, and (iv) Are phenotypic variation and genetic diversity clinally distributed along the precipitation gradient? We assessed these questions by measuring the thickness of both endosperm and of coat, as well as the endosperm to seed coat proportion (ESCP) in individuals of *T. officinale* from 25 populations (distanced among them for 1–1.5 km) and clustered in five localities (with five populations each one) distributed along a latitudinal rainfall gradient in Chile. Additionally, we evaluated the germination percentage in seeds from these five localities exposed to different amounts of water. We also evaluated the possible evolutionary change in seed traits and its adaptive consequences through two approaches: first, we evaluated the percentage of seeds germination of populations from the northern and southern limits of the gradient (La Serena and Coyhaique, respectively) through a cross-latitude experiment. Second, we conducted a reciprocal transplant experiment to assess the survival of adults and biomass accumulation with the same set of individuals from two localities (La Serena and Coyhaique). On the other hand, we estimated the heritability of ESCP by analyzing the linear relationship between the expression of this trait in maternal plants and their progeny. Additionally, we assessed the genetic differentiation among populations along the latitudinal gradient and also explored the relationships between genetic differentiation and geographic, trait, and environmental distances. Finally, since *T. officinale* often reproduces through asexual seed production (apomixis), we also used molecular markers to quantify the proportion of clones within populations, as among populations genetic and seed traits differentiation could be strongly affected by increased clonality due to this reproductive system.

## Materials and Methods

### Seed Collection

**FIGURE 1:**
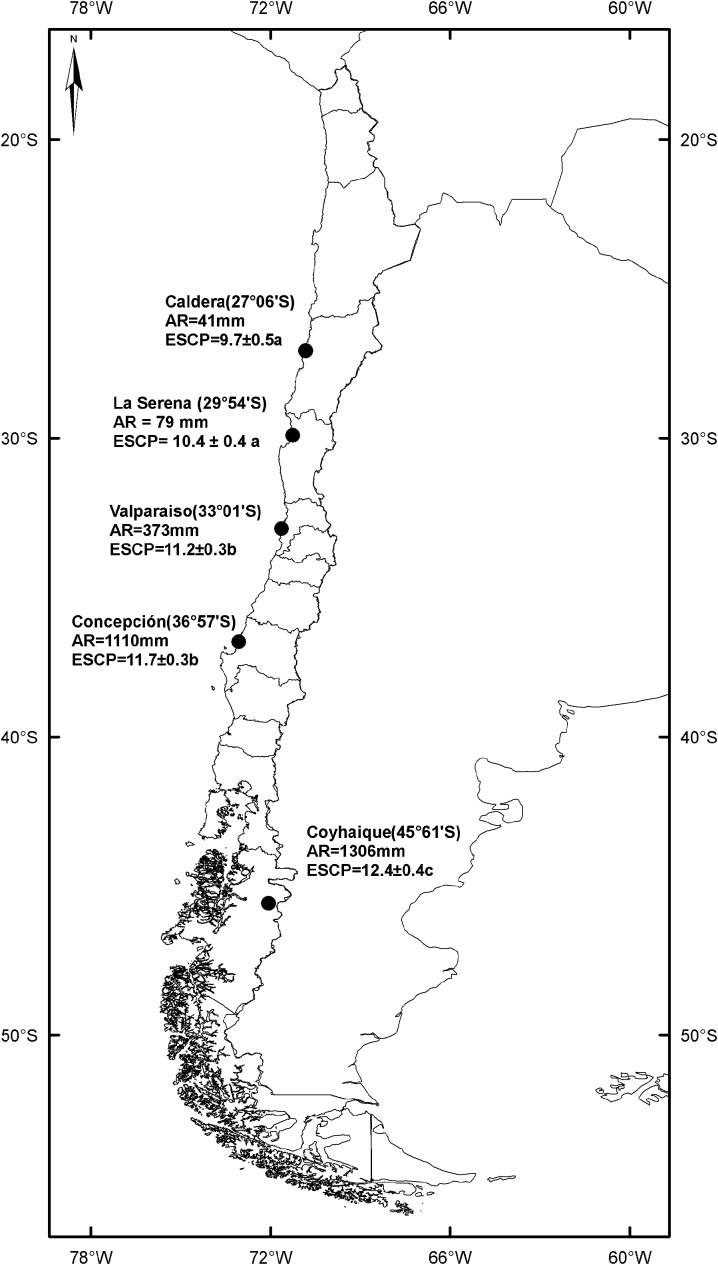
Map showing the five sampled localities along the latitudinal gradient. Annual rainfall (AR) and endosperm to seed coat proportion (ESCP) are given for each locality. Significant differences between ESCP values are denoted with different letters (*a posteriori* Tukey test α = 0.05).

Seeds of *Taraxacum officinale* Weber ex. F.H. Wigg. (Asteraceae) were collected in 25 populations from five different locations in Chile: Caldera, La Serena, Valparaiso, Concepción and Coyhaique. This broad latitudinal gradient covers from ca. 27°S to ca. 46°S, including a pronounced rainfall gradient that harbors a mean annual precipitation increase from 41 to 1306 mm in the sampling sites (**Figure 1**). All populations were separated at least by 1 km and at elevations between 0 and 50 m to reduce altitudinal effects. A range of 5–10 achenes (seeds hereafter) per individual was collected from 20 to 30 sampled plants per population to form the initial seed pool. As *T. officinale* has apomictic reproduction (production of seeds without fertilization), samples were taken from widely separated plants to capture a representative sample of phenotypic variation and genetic of each studied population. First generation plants (*F*_1_) were generated from this initial seed pool and were grown in a greenhouse at Universidad de Concepción, Concepción under natural conditions of light and temperature (day conditions_(08:00-19:59__h)_ = 978 μmol m^-2^ s^-1^ ± 85 and 18°C ± 3, and night conditions _(20:00-07:59__h)_ = 122 μmol m^-2^ s^-1^ ± 49 and 5°C ± 2). These plants were put in 300-mL plastic pots filled with potting organic soil and irrigated every 2 days with 50 ml of water. After 5 months these plants produced the achenes that were used to obtain experimental plants (*F*_2_).

### Seed Trait Analysis

We randomly selected five seeds from five different maternal plants (*F*_2_) in each locality (total *n* = 125 seeds) to measure the thickness of endosperm and coat, as well as the ESCP. Each seed was cut in half on the equatorial axis with a razor blade. Sectioned seeds were maintained with the section face up using modeling clay and photographed with a digital camera (Motic 3000 Cooled). Each picture was analyzed with specific software for analysis of digital images (Motic Images advance 3.2, Motic Chinese Group, Co., Ltd.). The computer coupled with the digitalization software gave the area and thickness of endosperm and coat of each seed. In addition, the ESCP was calculated as thickness of endosperm (endosperm plus embryo) relative to thickness of coat (seed coat plus achene wall), both in the equatorial section of seed. To assess the variation in the ESCP with latitude, a linear regression was conducted. In addition, differences in the thickness of in both endosperms and coat, and ESCP among localities were assessed with one-way ANOVA and Tukey’s HSD *post hoc* comparison test.

### Manipulative Experiment of Germination

Germination assays were carried out in order to test whether the structural trait analyzed here (ESCP) affected germination under different watering treatments. For each locality, 20 Petri dishes (100 × 15 mm) with 40 seeds each were prepared using two layers of wet filter paper (0.3 g/m^3^ of density) as substratum in order to avoid loss of humidity (total *n* = 4000 seeds). Five Petri dishes (one per population within each locality) were watered with 10, 25, 50, or 100 ml of distilled water on two occasions (*n* = 100 Petrie dishes). We selected these amounts of water because they represent typical conditions seeds might encounter in real conditions along the latitudinal rainfall gradient. All Petri dishes were watered once at the beginning of the experiment and placed in a growth chamber with 14/10 day/night photoperiod and 20 ± 1°C constant temperature. Dishes were covered with filter paper, and in order to maintain the differences in water availability for each watering condition, weight of each Petri dish was measured every 3 days throughout experiment. Germination was recorded daily until no new germinated seeds were observed for three consecutive days (day 30). The germination percentages among localities were compared with one-way ANOVA for each watering treatment after arcsine transformation and Tukey’s HSD *post hoc* comparison test.

### Cross-Latitude and Reciprocal-Transplant Experiments

#### Germination Experiment

We conducted a cross-latitude experiment using seeds collected from plants from five populations in La Serena (29°54′S) and five in Coyhaique (45°61′S) to investigate possible evolutionary change in endosperm to coat seed proportion and its adaptive consequences. At each of these populations we collected seeds from forty maternal plants and maintained them in paper bags. Forty seeds from each population (*n* = 200 per locality) were collected and bulked previous to buried at 1 cm of deep in plastic boxes (20 × 20 × 15 cm) arranged in the field. The cross-latitude experiment was conducted in both localities and with seeds from La Serena and Coyhaique. In each locality, we put 20 boxes with ten seeds each, of which half contained seeds from the La Serena, and the other half contained seed from the Coyhaique. In addition, we filled two plastic boxes with soil from each site without addition of seeds in order to assess the presence of *T. officinale* in the seed-bank. Each box was watered once with 10 ml in the locality of La Serena and with 100 ml in the locality of Coyhaique in order to mimic a typical rainfall of 2 days in each locality. Once per week, over 7 weeks, germination was recorded in each box. Germination percentage at 7 weeks of *T. officinale* individuals from both localities was compared by one-way ANOVA. Comparisons were conducted independently for each locality where individuals were transplanted.

#### Transplant Experiment

We conducted a reciprocal transplant experiment with plants originated of five populations from the two studied localities (La Serena and Coyhaique) to investigate possible evolutionary change in ESCP and its adaptive consequences. At each of these five populations in each locality, we collected seeds from 40 maternal plants, and germinated in a room with a photon flux density (PFD) of 470 (±135) μmol m^-2^s^-1^ at 20°C ± 2 in Petri dishes. Five days after the appearance of the first true leaf, seedlings were transferred to growth chamber at 10°C with a photon flux density (PFD) of 250 (±12) μmol m^-2^s^-1^ and 16/8 h light/dark photoperiod. This temperature was chosen because this is an intermediate of annual mean temperature of both localities (Molina-Montenegro et al., 2013). Ten 1-month-old seedlings from each population, each one from a different mother plant, were transplanted directly in a garden plot and randomly assigned following a grid design in both, La Serena and Coyhaique localities (*n* = 100 individuals from each locality). Each group of ten individuals (population) was separated by 1 m between each point of transplant. The plants were watered once every day only for the first week in order to reduce the death of plants due to the transplants. Once per week, over 10 weeks, survival was recorded for each plant maintaining the recording separated by locality of origin. All surviving plants were harvested after 10 weeks and total plant biomass was obtained after the whole plants were oven-dried at 70°C for 72 h. Considering that neither survival percentage [one-way ANOVA = *F*_1,8_ = 24,56; *p* = 0.65 and *F*_1,8_ = 16,33; *p* = 0.75 for La Serena and Coyhaique, respectively] nor biomass [one-way ANOVA = *F*_1,8_ = 41,56; *p* = 0.71 and *F*_1,8_ = 79,13; *p* = 0.21 for La Serena and Coyhaique, respectively] differed between populations from the same locality of origin, we merged the results by locality. Survival percentage and biomass at 10 weeks of *T. officinale* individuals from both localities were compared by one-way ANOVA (STATISTICA 7.0). Comparisons were conducted independently for each locality where individuals were transplanted.

### Heritability of ESCP

We estimated the heritability by analyzing the relation between the ESCP of maternal plants and the same trait in their progeny (parent–offspring regression method, *sensu* Falconer, 1981). It has been shown that seed traits like seed pubescence, shape and thickness have significant heritability (Gómez-González et al., 2011), which might imply a genetic and evolutionary response with probability to change from generation to generation, as classically predicted by the product of the selection index and the heritability estimate (Falconer, 1981).

We focused on the same populations of the germination experiment. From the previous seeds collected in the field in each of 25 populations we generated new plants (*F*_2_) in order to clean of “maternal effects.” From this second generation, five maternal plants per population were obtained in order to produce the experimental progeny (*F*_3_). Seeds were germinated in Petri dishes and 2-week-old seedlings were planted in pots with a 1:1 sand:commercial soil mixture. All pots were watered every 3 days at field capacity. Plants were grown in a common environment in a greenhouse until flowering (PFD of 750 μmol m^-2^s^-1^± 310, 50 cc of tap-water per pot every 2 days, and at 24°C ± 5). Although *T. officinale* has been described as an apomictic species, it is also self-incompatible (Jenniskens, 1984). Seed set also occurs through cross-pollination and hence, pollinator exclusion were needed to accurately estimate the heritability of the ESCP. Therefore, heads were protected from pollinators with a fine, transparent mesh bag (15 × 10 cm) with a pore size of 0.9 × 0.9 mm until seed maturation (70-95 days). ESCP of three seeds in three different maternal plants per population and from three seeds (as defined previously) from three *F*_3_ plants per population were measured (total *n* = 225 seeds per mother-progeny group) and compared by a regression analysis (Zar, 1996).

### Genetic Diversity and Population Structure

Total DNA was extracted from dry foliar tissue of plants previously assessed in each locality according to cetyl-trimethyl-ammonium bromide (CTAB) method (Doyle and Doyle, 1987), following the protocol described in Cota-Sánchez et al. (2006). Final DNA concentrations were quantified in a NanoDrop^®^ spectrophotometer (ThermoFisher, United States), and their integrities verified by electrophoresis in a 1% agarose gel. The genetic diversity and population structure were estimated using Amplified Fragment Length Polymorphism (AFLP). The AFLP protocol was performed following the description of Vos et al. (1995). Genomic DNA (∼250 ng) was digested in a total volume of 25 μL using EcoRI (NEB) and MseI (NEB) restriction enzymes (1U each) for 2 h at 37°C, followed by 15 min at 70°C. The resulting DNA fragments were then ligated with the corresponding EcoRI (5 pmol) and MseI (50 pmol) adapters using T4 DNA ligase (1U) and 1 × ligation buffer (Roche) for 3 h at 37°C. Preselective amplification reactions were performed using 1 μL of digested-ligated DNA in a total volume of 20 μl. The mixture contained 1 × PCR buffer, 1.5 mM MgCl_2_, 0.25 mM of each dNTP, 0.5 μM Eco + A primer, 0.5 μM MseI + C primer and 1U of AmpliTaq Gold^®^ DNA polymerase. PCR amplification was carried out with a profile of 20 cycles of denaturation 30 s at 94°C, annealing 1 min at 56°C, and extension 1 min at 72°C. After PCR amplification, amplification products were diluted 1:10 with distilled water and stored at -10°C. Selective amplifications were performed using a 1 μL of diluted preselective amplification as a template in reactions containing 1x PCR buffer, 1.5 mM MgCl_2_, 0.25 mM of each dNTP, 0.5 μM Eco + ANN (fluorescent) primer, 0.5 μM Mse + CNNN (or Mse + CNNN) primer, and 1U of Platinum Taq DNA polymerase (Invitrogen Brazil). Selective amplifications started with a touchdown step of 2 min at 94°C, then 10 cycles of 30 s at 94°C, 30 s at 65°C (1°C decrease each cycle) and 1 min at 72°C. This was followed by 30 cycles of 30 s at 94°C, 30 s at 56°C and 1 min at 72°C, ending with a final elongation of 30 min at 72°C.

Following Majesky et al. (2012), we used three selective primers combinations: E + AGC/Mse + CAAT, Eco + AAT/Mse + CAAC and EcoAGC/Mse + GATG that produced 85, 61 and 50 fragments, respectively. All profiles were run in an automatic sequencer (Applied Biosystems 3120, 16 capillaries) at the Molecular Biology Laboratory of the Pontificia Universidad Católica de Chile. For each pair of primers combination, fragments with sizes between 80 and 450 bp, and intensities > 200, were first selected using GeneMarker 2.4.0 (Soft Genetics). These fragments were coded in a presence/absence (1/0) matrix. The chosen fragments were, besides polymorphic, present in no less than 5% (nor more than 95%) of the individuals, and error rates of replication were lower than 8%. AMaRe (AFLP Matrix Reduction) method, outlined in Kück et al. (2012), was used to calculate reliability and error rate of the matrix. The average unbiased expected heterozigosity (*H*_E_), percentage of polymorphic loci (% PL; a loci was considered polymorphic if at least in 5% of the sample the less frequent state was observed) and Shannon’s index of diversity (*I*) were estimated using GENALEX (Peakall and Smouse, 2006).

Population genetic structure was investigated though Discriminant Analysis of Principal Components (DAPC) using the *adegenet* R-package (Jombart, 2008; Jombart et al., 2010), and hierarchical analysis of molecular variation (AMOVA). First, DAPC analysis was applied to the AFLP data to visualize the potential clustering of individuals, which together with the pair-wise *F*_ST_ values between localities, was used as the criteria to define a population structure to be tested in the analysis of molecular variance (AMOVA). Both, *F*_ST_ and AMOVA analysis were performed in Arlequin 3.5 (Excoffier et al., 1992), all other analysis were carried out on the R language and environment for statistical computing v3.1.3 (R Core Team, 2015).

We explored the relationships between genetic differentiation among localities (pairwise *F*_ST_) and geographical distance (Euclidean), differences in mean annual rainfall and differences in seed coat to endosperm proportion. The level of correlation between pairs of distance matrices was estimated using Mantel test’s (Mantel, 1967). The significance for each correlation coefficient between matrix pairs was obtained from the distribution of 9999 randomizations as supported by the “ade4” R-package (Dray et al., 2007).

### Clonality Assessment

Asexual reproduction (agamospermy) is common in dandelions (Asker and Jerling, 1992). Thus, in its native range of distribution natural populations can be formed either by co-occurring sexual diploids and apomictic triploids, or by exclusively by apomictic triploids (Asker and Jerling, 1992; van der Hulst et al., 2000; Majesky et al., 2012). To assess whether the studied populations are formed by few successful clonal lineages or by several multi-locus lineages, we estimated the extent of clonal reproduction in our AFLP dataset using the software GenoDive 2.0b17 (Meirmans and Van Tienderen, 2004). We choose GenoDive because it has been extensively employed to estimate clonal diversity on marine organisms (e.g., Blouin and Brawley, 2012) and plants (e.g., Lasso, 2008; de Witte et al., 2012) using AFLP markers. Briefly, GenoDive uses a “threshold” of genetic distance between pairs of individuals to distinguish clones from non-clones. Below that threshold of genetic dissimilarity, samples are considered to represent a single clone. In other words, this threshold indicates the maximum distance that is allowed between a pair of individuals to still be considered clonemates (individuals from the same clonal lineage). GenoDive assumes that random mating within populations, i.e., its tests whether the allelic frequencies deviate from these that are expected under random mating. To do this, GenoDive tests the null hypothesis that the observed clonal diversity is due to sexual reproduction by randomizing alleles over individuals and comparing the observed clonal diversity with that of the randomized dataset uses (Gomez and Carvalho, 2000). It is important to note that other processes than asexual reproduction such as self-fertilization can also lead to identical genotypes, and therefore, under random mating clones can also be produced. Since scoring errors and mutations may cause individuals from the same clonemate to have a pairwise distance larger than zero, choosing an appropriate threshold is crucial to perform accurate clonal assignments (Rogstad et al., 2002; Lasso, 2008). While too low thresholds overestimates the estimates of clonal diversity, choosing this value too high results in its underestimation.

Our dataset consisted of a total of 89 individuals from 5 populations genotyped with AFLP’s (Supplementary Table S1). To reduce the scoring error rates in clone assignment we previously eliminated from our dataset all loci containing missing genotypes (45 loci). In addition, scoring errors were checked using the software AMARE (Kück et al., 2012), This analysis revealed that scoring error rate was reduced from 5% in the total data set (196 loci) to 2% in the subset used to estimate the levels of clonality (151 loci). After this procedure we selected this subset of 151 AFLP bands to evaluate clonal diversity. Specifically we made a clone assignment analysis to determine the number of multilocus lineages (MLLs) within each population and the clonal fraction (i.e., the ratio of MLLs to samples) which was calculated as (*N* – MLLs) / *N*. The analyses were run considering the following parameters: (i) a threshold of 6 loci (i.e., pairs of individuals that differed in a maximum of 6 loci were considered as clonemates), (ii) an infinite allele model, (iii) 1,000 permutations and (iv) randomizing of alleles over individuals within populations, and over individuals over all populations. Since most studies to date have reported threshold values between 2 and 4% (e.g., van der Hulst et al., 2000; Douhovnikoff and Dodd, 2003; Douhovnikoff et al., 2004), we used a threshold of 6 for our dataset as it represents a 4% (6/151 loci = 0.039) of AFLP genetic dissimilarity between pairs of individuals.

## Results

### Seed Trait Analysis

**FIGURE 2:**
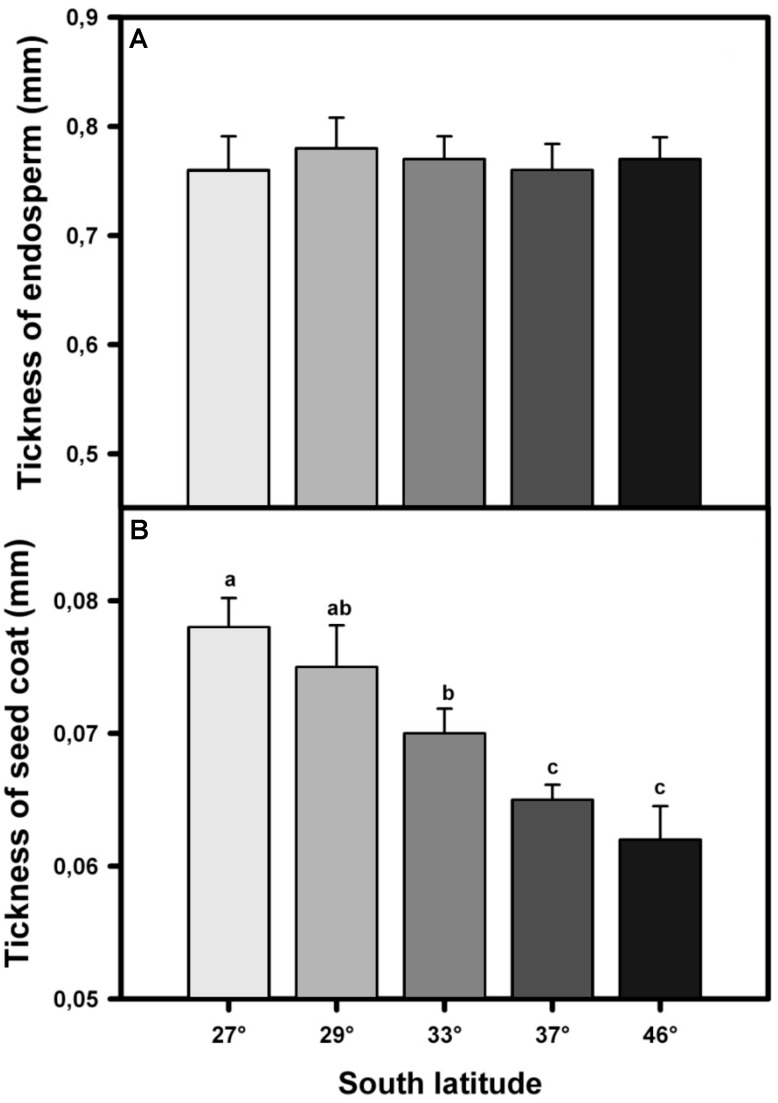
Mean values (±2 SE) of thickness of endosperm **(A)** and seed coat **(B)** in seeds of *Taraxacum officinale* collected from five localities along a latitudinal gradient. Significant differences between localities are denoted with different letters (*a posteriori* Tukey test α = 0.05).

The ESCP showed a strong and significant positive relationship with mean annual precipitation (*r*^2^ = 0.95; *p* < 0.01, **Figure 1**). Contrarily, the thickness of endosperm (endosperm plus embryo) was not different (*F*_4,120_ = 11.17; *p* = 0.79) between seeds of individuals from all localities (**Figure 2A**). On the other hand, seed coat thickness showed a significant variation with latitude, with *T. officinale* from northern populations having a thicker seed coat than those from southern populations (*F*_4,120_ = 553.43; *p* < 0.001, **Figure 2B**).

### Manipulative Experiment of Germination

**FIGURE 3:**
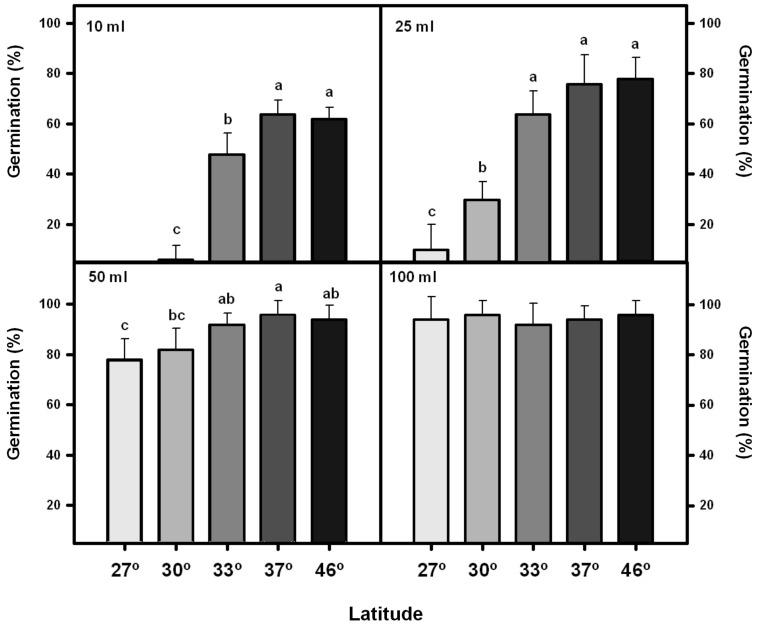
Germination percentage of *Taraxacum officinale* seeds collected from five localities along a latitudinal gradient and exposed to different amounts of water irrigation treatments. Mean values (±2 SE) in germination of five populations of every locality along the latitudinal gradient are shown. Significant differences between localities are denoted with different letters (*a posteriori* Tukey test α = 0.05).

Germination percentage increased considerably in the treatments with greater addition of water (**Figure 3**). Seeds from southern localities showed significantly higher percentage of germination (*F*_1,4_ = 32.53; *p* = 0.02) than those from the northern ones (**Figure 3**). This trend was more pronounced in the treatment with a lower water addition (10 ml: *p* < 0.001; 25 ml: *p* < 0.001; 50 ml: *p* < 0.01; 100 ml: *p* = 0.88).

#### Cross-Latitude and Reciprocal-Transplant Experiments

**FIGURE 4:**
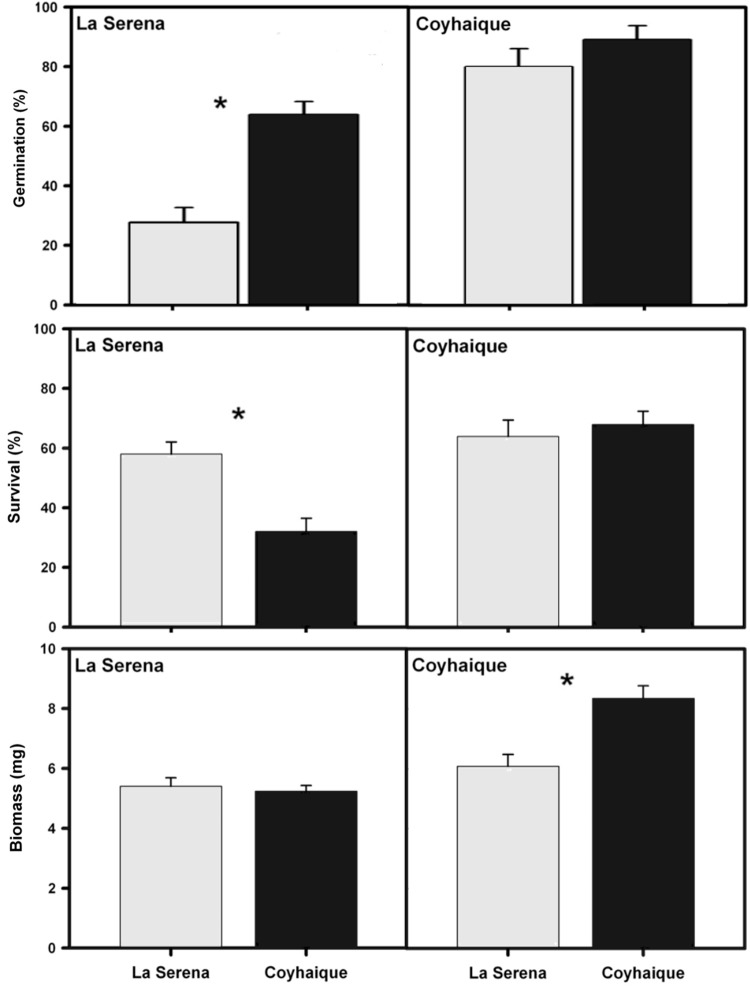
Mean values (±2 SE) of germination percentage, survival percentage and total biomass (mg of dry weight per plant) of *T. officinale* individuals from La Serena and Coyhaique and put in both La Serena and Coyhaique. The locality name in the upper-left corner of each box denotes the place where transplants were made. Asterisk indicates significant differences.

Overall, the germination experiment showed that germination percentage was higher in those seeds with slimmer seed coat and in those located in Coyhaique. Specifically, seeds produced by *T. officinale* individuals from Coyhaique had significantly higher germination percentage (*F*_1,8_ = 64.35; *p* < 0.001) than those from La Serena when they were buried in La Serena (**Figure 4**). In contrast, seeds of *T. officinale* individuals from both localities when were buried in Coyhaique showed similar germination percentage (*F*_1,8_ = 2.57; *p* = 0.29, **Figure 4**).

On the other hand, the reciprocal transplant experiment showed that both survival and biomass were similar among populations within each origin but differed between La Serena and Coyhaique (**Figure 4**). Specifically, *T. officinale* individuals from La Serena had significantly higher survival percentage (*F*_1,16_ = 108.1; *p* < 0.001) than those from Coyhaique (**Figure 4**). Interaction between site of transplant and origin of plants (S × O) was significant (*F*_1,16_ = 241.8; *p* < 0.001), since individuals of Coyhaique had lower survival than those from La Serena but only when they were transplanted in La Serena (**Figure 4**). In addition, the biomass accumulation in *T. officinale* individuals from both origins was significantly greater when were grew-up in Coyhaique (*F*_1,16_ = 91.1; *p* < 0.001; **Figure 4**). Interaction (S × O) was significant (*F*_1,16_ = 5.7; *p* < 0.001), since individuals of Coyhaique had higher biomass accumulation than those from La Serena but only when they grew-up in Coyhaique (**Figure 4**).

#### Heritability of Seed Trait

**FIGURE 5:**
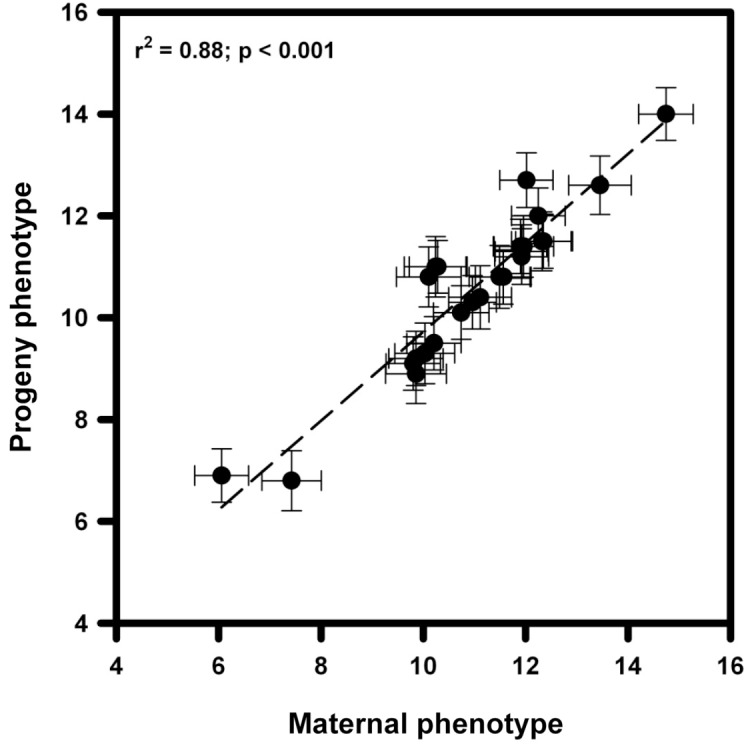
Heritability of the ESCP in *Taraxacum officinale* seeds from 25 populations belonging to five localities distributed along a latitudinal gradient. Means of the relationship between the maternal phenotype and the offspring phenotype of nine seeds per population are shown.

The expression of the ESCP showed a significant positive relationship between maternal plants and their progenies (*r*^2^ = 0.88; *p* < 0.001), suggesting that this trait is heritable and has the potential to evolve by natural selection (**Figure 5**).

#### Genetic Diversity and Relationships with Environmental Variables

Overall, the levels of within-locality genetic diversity were moderate. The mean unbiased expected heterozigosity (*H_e_*) was 0.183 (±0.007), ranging from 0.129 (±0.014) in Valparaiso to 0.229 (±0.015) in Caldera. Similarly, the mean percentage of PL was 52.4%, ranging from 39.2% in Valparaiso to 71.3% in Caldera. None of these estimators were correlated with the environment, latitude or seed traits (**Table 1**).

**Table 1:** Summary of Mantel correlation coefficients between the studied seed trait (endosperm to coat proportion) and environmental, genetic, and geographic distance matrices among populations.

vs.	Genetic	Geographic	Environmental	Trait
Genetic	–			
Geographic	0.32^ns^	–		
Environmental	0.50^ns^	0.76^∗∗^	–	
Trait	0.55^ns^	0.82^∗^	0.74^∗^	–

*The significance of the standardized Mantel statistic was tested by permutation. ^*ns*^p > 0.05; ^∗^p < 0.05, ^∗∗^p < 0.01*.

**FIGURE 6:**
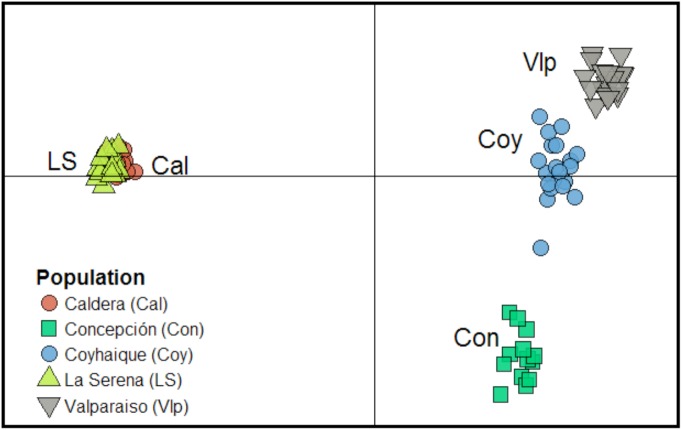
Results of the DAPC analysis over the 196 AFLP loci genotyped for 90 *T. officinale* individuals. Samples from five localities are included (Caldera, La Serena, Valparaiso, Concepción and Coyhaique) along the latitudinal gradient. The first two discriminant functions show that most genetic variation is explained by four clusters, with a clear divergence between northern and southern populations.

The Discriminant Analysis of Principal Components (DAPC) analysis revealed four clusters with a major disruption between north-south localities (**Figure 6**). In a mixed cluster the northern localities of Caldera and La Serena denote their close genetic relationship, but also a strong differentiation from the rest of individuals. In the other hand, individuals from the more southern localities of Valparaiso, Concepcion and Coyhaique, albeit each one appeared as isolated groups, they are all opposed to the northern localities (**Figure 6**). Consistently, genetic differentiation (pairwise *F*_ST_) between Caldera and La Serena was not significantly different from zero, averaging 0.56 and ranging from 0.31 between Valparaiso and Coyhaique localities, to 0.82 between Valparaiso and La Serena (**Figure 6**). A four-localities structure, following the DAPC and *F*_ST_ results, was proposed for testing in the analysis of molecular variance (AMOVA): Valparaiso, Concepción and Coyhaique as individual groups, and Caldera and La Serena joined in the fourth one. This analysis revealed a strong locality structure (*F*_ST_ = 0.614, *p* < 0.001) with the remaining ∼38% of the observed variation as within-locality variation.

**FIGURE 7:**
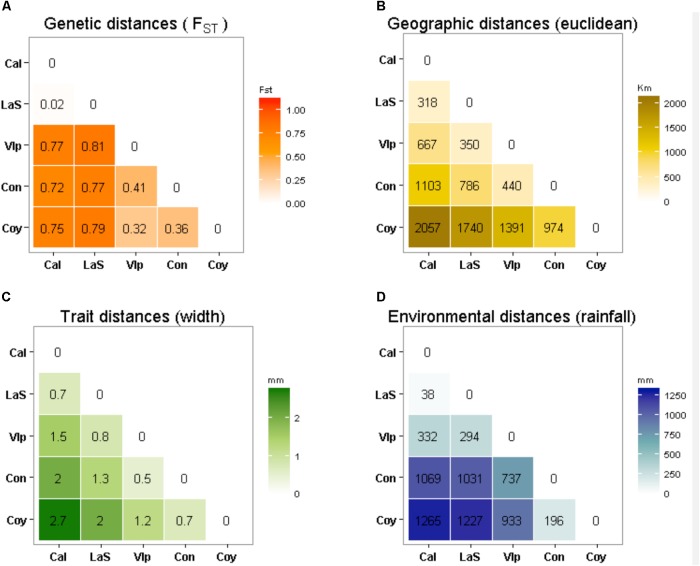
Matrix of genetic **(A)**, geographic **(B)**, trait **(C)** and environmental **(D)** distances between *T. officinale* individuals from five localities (Caldera, La Serena, Valparaiso, Concepción and Coyhaique), along the latitudinal gradient. With the exception of the pairwise *F*_ST_ value between Caldera and La Serena (*F*_ST_ = 0.002), all other genetic distances were statistically significant (*p* < 0.05).

Finally, despite high levels of correlation were found between the geographical, environmental and trait distance matrices among localities (**Figure 7**), there was no relationship between any of them and the matrix of pairwise genetic distances, as showed by the significance of the Mantel test coefficients (**Table 1**). Nevertheless, the Mantel correlation (*r*M = -0.69, *p* = 0.074) suggest an interesting trend between the genetic and the phenotypic traits (**Figure 7**).

### Clonality Assessment

Considering all populations, 75 out of the 89 samples were unique multi locus lineages (MLLs) Thus, the average clonal fraction (for the entire dataset) was 0.16, ranging from 0.11 in Caldera to 0.20 in Coyhaique (Supplementary Table S1). Since clones were found in all populations, the hypothesis of random mating was not met (Supplementary Table S1). The number of clone pairs in the entire dataset was 11, ranging from 1 in Valparaiso to 3 in La Serena and Coyhaique (Supplementary Table S2). Although apomixis (clonal reproduction through seeds) is an important mode of reproduction in dandelions, our results indicate asexual and sexual reproduction in Chilean dandelions. This result is consistent with the findings of previous studies addressing the importance of asexual and sexual reproduction in *T. officinale*. For instance, van der Hulst et al. (2000) studying a single population of triploid dandelions from Jutland (Denmark) found that, although asexual reproduction (apomictic) result in overrepresented widespread genotypes, sexual recombination has substantial contribution to genetic variation. Interestingly, the clonal fraction found by van der Hulst et al. (2000), was greater (0.59) than that found in this study (0.16), suggesting higher rates of sexual reproduction in Chilean dandelions. In a recent study, Majesky et al. (2012) found low levels of genotypic diversity (high proportion of clones) in European dandelions. However, they also found evidence of mutation and sexual recombination, suggesting that clones can be originated from hybridization between sexual (diploids) and apomictic (triploids). Therefore, clonality analyses indicate that Chilean populations of dandelions consist of multiple clones, and there is substantial genetic variation for natural selection act upon.

## Discussion

Here, we show evidence of adaptation and heritability in seed coat to endosperm proportion of the seeds of the invasive *T. officinale* that suggests a rapid differentiation of this plant species into ecotypes adapted to different rainfall levels. This may help to explain why *T. officinale* can successfully colonize broad spatial range with contrasting environmental conditions.

Most of studies that have assessed key invasiveness traits have compared the fitness and functional traits between invasive and native species (van Kleunen et al., 2010; Godoy et al., 2011; Molina-Montenegro et al., 2012b, 2013). In contrast, differences in plant performance among population within non-native range have received much less attention, despite the fact that genetic variation may be critical for invasiveness potential (Meyer and Allen, 1999; Neuffer and Hurka, 1999; Vellend et al., 2010; Molina-Montenegro et al., 2011, 2013). Recent literature has highlighted the evolutionary dimension of biological invasions (e.g., Bossdorf et al., 2005; Thorpe et al., 2011; Colautti and Lau, 2015). However, the discussion on how local adaptation influences the spread of invasive species has been highly speculative, as it is based on a limited number of quantitative studies (but see García-Ramos and Rodríguez, 2002; Urbanski et al., 2012; Colautti and Barrett, 2013; Molina-Montenegro et al., 2010, 2013; Colautti and Lau, 2015). Our results show empirical evidence about the variation in a seed trait with adaptive value in an invasive plant species. This information could be used for management of invasive species and for the identification of potential new invaders. In fact, some studies have identified several traits related with physiology, environmental tolerance, fitness and/or life-history traits, higher represented in invasive species than natives one (Pysek and Richardson, 2007; Molina-Montenegro et al., 2012a). Thus, to recognize several traits (e.g., those related with seeds) that enhance the environmental tolerance, survival or spread capacity, could be an early indicator to estimate the potential of invasion for a given set of species.

Several biological studies (reviewed in Phillips et al., 2006) have documented that classical models may underestimate the speed of invasion process, suggesting that evolutionary responses to novel environments might speed up invasions. Namely, the evolution of morphology and life-history traits can have strong impact on the invasion success and expansion of invading species (Phillips et al., 2006; Molina-Montenegro et al., 2012a; Hulme and Barrett, 2013; Pysek et al., 2015). For example, Maron et al. (2004) found a clinal variation in several morphological and functional traits in the introduced populations of *Hypericum perforatum*, suggesting that these variations can help explain the quick spread of this invasive species in North America. In this context, the study of traits involved in the adaptation to a rainfall gradient, such as variation in seed coat thickness found in *T. officinale*, is pivotal to understand the adaptation and evolution of invasive plant species.

In seminal studies of biological invasions, evolutionary biologists postulated that genetic variation and adaptive evolution through natural selection are involved in the success of invading species (Baker, 1965; Mayr, 1965). A growing number of studies have shown that putatively adaptive traits have evolved in introduced populations (Rogers and Siemann, 2004; Molina-Montenegro et al., 2011; Colautti and Lau, 2015), sometimes quite rapidly (e.g., less than 10 years) (Reznick and Ghalambor, 2001; Leger and Rice, 2007; Whitney and Gabler, 2008). Continuous spread of *T. officinale* has probably taken place since the first recorded occurrence in 1870 and even that some populations are far from this city, it seems unlikely that this species has been present for more than 50 years in all localities considered here. Notwithstanding of this short period of time, some of these populations have shown a significant differentiation in the mean value of the assessed seed trait among *T. officinale* individuals from the native range and the invaded localities in Chile. For instance, seeds of *T. officinale* individuals collected in the native range (2600 m a.s.l., showed an endosperm to seed coat proportion = 12.2 ± 0.3; endosperm = 0.76 ± 0.11 mm; and seed coat thickness = 0.062 ± 0.06 mm). These values are similar to those found in the southern populations of Chile, where the annual rainfall is similar to the native range (annual rainfall = 1560 mm, Molina-Montenegro et al., 2011), but quite different of those populations in Chile with contrasting rainfall amounts. Thus, our results suggest that individuals of *T. officinale* arrived to Chile had to adapt several functional traits (e.g., seed coat) according the climate conditions experienced in each locality in order to germinate and establish permanent populations.

The high variability in the expression of the seed traits assessed among the Chilean *T. officinale* populations appeared to be highly related to experienced variation in rainfall. But moreover, as they also show evidence for genetic differentiation and heritability in those traits, the potential to adapt to local environmental conditions and thus evolve in their new habitats rapidly, is not rule out for this species. This could be observed in the two northern locations, Caldera (27°05′S) and La Serena (29°54′S), despite being 402 km apart, their similar extremely dry conditions seem to greatly influence the genetic composition of *T. officinale* individuals. Currently, similar genotypes are present in both localities (pair-wise *F*_ST_ = 0.002) such as to consider them as belonging to the same population (Frankham et al., 2002), even when both present high levels of genetic diversity (71 and 54% of PL, respectively). Interestingly, at a similar distance (439 km) toward the south, in Valparaiso (33°02′S), significant differences are already observable in both, seed traits and genetic composition. In this sense, the most evident genetic disruption seems to occur between the two northern populations and the rest of individuals. This may suggest that the ∼371 mm of Valparaiso’s rainfall, in comparison with ∼41 mm and ∼79 mm of Caldera and La Serena, could be enough to relax the environmental filter that maintain the genetic similarity within the northern individuals of *T. officinale*. Indeed, all southern locations appeared genetically differentiated between them; however, the spatial pattern for their genetic divergence was not latitudinally oriented, suggesting that genotypes are only partially linked to the clinal variation observed in the seed structure among the studied populations of *T. officinale*. Nevertheless, is not possible discard that different historical patterns of introduction can explain these differences also. In fact, the history of introduction in Chile suggests more than only one event did occur; even these introductions appear to be simultaneously in many localities spatially distanced. Thus, it is possible to propose two alternative hypotheses: first, individuals of *T. officinale* arrived to new range and once established, the environment did act as selective pressure, filtering some traits (e.g., seed coat), favoring individuals with thicker and thinner seed coat in driest and wetter sites, respectively. Second, *T. officinale* was introduced repeatedly from multiple origins, and only those genotypes that were pre-adapted to the new environment could maintain themselves over time. For example, those genotypes in the northern populations of Chile may have received propagules from a dry locality in its native range, or even from other dry sites of the non-native range (e.g., California). However, the high genetic similarity between the populations of these two localities suggests that they were originated from the same colonization event. Future studies should address the hypothesis of multiple introduction events of *T. officinale*, by mean of phylogeographic approaches, to understand the evolutionary processes of differentiation in its introduced range (see, Leger and Rice, 2007).

Rapid adaptive evolution has been advocated as an important mechanism involved in successful invasion by exotic plant species (Maron et al., 2004; Sax et al., 2007; Cano et al., 2008; Colautti and Lau, 2015). When species are introduced into a new region they may face novel selective regimes, under which, shifts in genetically based phenotypic traits may increase the fitness of individuals (Suarez and Tsutsui, 2008). Convincing evidence of rapid adaptive evolution has been reported in some invasive animals (Phillips et al., 2006; Urbanski et al., 2012), but such evidence has been seldom documented in invasive plants (but see, Barrett et al., 2008). Here, we found a likely example for adaptive evolution in an invasive plant species, where the thickness of seed coat is modified for changes in precipitation, affecting the percentage of germination and enhancing the probability of seedling survival in *T. officinale* according to the abiotic conditions of each population. Thus, if fitness-related traits measured in *T. officinale* are driven by differences in endosperm to seed-coat proportion and seed coat thickness, these differences may be an adaptive response due to differential resource allocation and fitness in different localities, or may potentially reflect divergent natural selection (*sensu* Haig and Westoby, 1990). Reciprocal transplant experiment indicates that seed coat thickness, have a strong adaptive effect, because they maximize the survival in arid environments and improve growth in moist environments, having a thicker and slimmer seed coat respectively. Hence, the rapid adaptive evolution and high heritability of ESCP allow *T. officinale* to successfully colonize and spread in habitats with different environmental conditions. Nevertheless, this capacity could be also related to other functional seed traits not analyzed in this study, for which it remains to be tested.

Considering the apomictic (asexual reproduction through seeds) reproductive mode of *T. officinale*, any relevant trait for plant fitness might be quickly fixed in stressful environments. This trend is evidenced in our results, where individuals from northern (dryer) population showed thicker coated seeds than those from wetter southern populations, and this seed trait was correlated with higher fitness when were sown in site with abiotic conditions similar to those found in their origin as showed by survival experiments. Thus, seeds with thicker coat will be successful in sites with low precipitations in order to avoid germination when abiotic conditions are not suitable. Precipitation here are scarce and seeds will need to germinate only when a certain amount of water is available, avoiding high seedling mortality. On the other side, populations growing in the southern sites produced seeds with a slim seed coat since with water availability for a good germination and early seedling growth is not limiting. The adaptive value of seed coat thickness was highlighted when the germination percentage of seeds from rainy population was significantly higher than the driest population, but the mortality was more pronounced in the rainy population. On the other hand, although both populations showed high germination percentage when sown in moist environment, those from the rainy sites showed significantly higher growth than dryer sites. Thus, ours results suggest that the clinal variation in the seed coat thickness of *T. officinale* populations has an adaptive value, positive impacting colonization and invasion of broad environmental gradients.

Alternatively, broadly distributed plants species must deal with a wide range of environmental conditions and respond to challenges imposed by environmental conditions by means of phenotypic plasticity (Valladares et al., 2014). Ecological theory predicts that phenotypic plasticity should be one of the main adaptive mechanisms in heterogeneous or changing environments as those found along a latitudinal gradient. In fact, the relative importance of phenotypic plasticity in morpho-physiological and fitness-related traits to explain the ample ecological breadth in *T. officinale* has been previously addressed (Molina-Montenegro et al., 2010, 2012a, 2013, 2018). Nevertheless, in the present study we discard the plasticity as a key factor to explain the current patterns in the seed trait assessed. First, previous to began the laboratory and field experiments we “clean” the maternal effects using seeds from a second generation (*F*_2_) and to assess the level of heritability we used a third generation (*F*_3_). In both cases, all “maternal plants” were growth under similar conditions but its seeds maintained the differences in the morphologic characteristics, suggesting the at least the seed-coat thickness is a not plastic trait.

Since rainfall regimes are changing worldwide due to global change processes (IPCC, 2013), it is essential that we understand how plant populations can respond to temporally variable conditions and rapidly adapt and colonize new environments. In a global change scenario, new environments would be available for shift or expand the native and/or introduced range by part of species that have the capacity to adapt rapidly. *T. officinale* seems to have a successful strategy that results in a great invasive potential. It seems that this invasive plant could likely evolve into specialized ecotypes reaching zones with contrasting climatic conditions. We are still far from comprehending the full process of invasion and the associated putative evolutionary process, nonetheless assessing more species or performing integrative analysis (e.g., meta-analysis) we might find promising approaches to better understand the role of evolutionary processes in invasion.

## Author Contributions

MM-M, CT-D, IA-R, and CA designed the experiments. MM-M, RH, AL, and TF performed the experiments. MM-M, CT-D, IA-R, RH, and TF analyzed the data. MM-M wrote the paper along with CT-D, IA-R, and CA. All authors reviewed the manuscript.

## Conflict of Interest Statement

The authors declare that the research was conducted in the absence of any commercial or financial relationships that could be construed as a potential conflict of interest.

## Footnotes

1 https://www.udec.cl
