# Supplementary material for: Is the Success of Plant Invasions the Result of Rapid Adaptive Evolution in Seed Traits? Evidence from a Latitudinal Rainfall Gradient
Source: Front Plant Sci. 2018 Feb 27;9:208. doi: 10.3389/fpls.2018.00208 (PMC5835042; doi:10.3389/fpls.2018.00208)
Supplement: Supplementary file 1 [file Table_1.docx]

**Table S1** Descriptive statistic of the genetic diversity parameters in the studied localities

| **Locality** | ***n*** | **MLL** | **T** | **Clonal Fraction** | ***p*** |
| --- | --- | --- | --- | --- | --- |
| Caldera | 19 | 17 | 6 | 0.11 | 0.001 |
| La Serena | 18 | 15 | 6 | 0.17 | 0.001 |
| Valparaiso | 19 | 16 | 6 | 0.16 | 0.001 |
| Concepción | 13 | 11 | 6 | 0.15 | 0.001 |
| Coyhaique | 20 | 16 | 6 | 0.20 | 0.001 |
| **All localities** | **89** | **75** | **6** | **0.16** | **0.001** |
| *n* = number of individuals; MLL = number of detected multilocus lineages (MLLs); T = threshold for multilocus genotype; Clonal fraction = (*n*-clones) ⁄ *n*; *p* = probability that sexual recombination is responsible for the repeated genotypes assigned to a MLL (calculated from the effective number of genotypes). | | | | | |

**Table S2** List of pairs of clones found in each sampled locality

| **Locality** | **Clone pair #1** | **Clone pair #2** | **Clone pair #3** |
| --- | --- | --- | --- |
| Caldera | Cal06 + Cal18 | Cal07 + Cal19 | – |
| La Serena | LS07 + LS17 | LS08 + LS18 | LaS13 + LaS14 |
| Valparaiso | Vlp02 + Vlp13 | – | – |
| Concepción | Con02 + Con04 | Con07 + Con12 | – |
| Coyhaique | Coy01 + Coy02 | Coy13 + Coy14 | Coy16 + Coy17 |
